# Ozone-Induced Biochemical and Molecular Changes in *Vitis vinifera* Leaves and Responses to *Botrytis cinerea* Infections

**DOI:** 10.3390/antiox12020343

**Published:** 2023-01-31

**Authors:** Margherita Modesti, Alessandra Marchica, Claudia Pisuttu, Samuele Risoli, Elisa Pellegrini, Andrea Bellincontro, Fabio Mencarelli, Pietro Tonutti, Cristina Nali

**Affiliations:** 1 Department for Innovation in Biological, Agro-Food and Forest Systems, University of Tuscia, Via S. Camillo de Lellis, 01100 Viterbo, Italy; 2 Department of Agriculture, Food and Environment, University of Pisa, Via del Borghetto 80, 56124 Pisa, Italy; 3 University School for Advanced Studies IUSS, Piazza della Vittoria 15, 27100 Pavia, Italy; 4 CIRSEC, Centre for Climate Change Impact, University of Pisa, Via del Borghetto 80, 56124 Pisa, Italy; 5 Crop Science Research Center, Scuola Superiore Sant’Anna, Piazza Martiri della Libertà 33, 56127 Pisa, Italy

**Keywords:** reactive oxygen species, oxidative burst, systemic acquired resistance, multiple-stress, grapevine, phytohormones, expression of resistance-related genes

## Abstract

To investigate how plants cope with multi-stress conditions, we analyzed the biochemical and molecular changes of *Vitis vinifera* leaves subjected to single or sequential double stresses (infection by *Botrytis cinerea* (*Bc*) and ozone (O_3_, 100 ppb for 3 h) treatment). In Bc^+^/O_3_^−^ leaves, the hydrogen peroxide (H_2_O_2_) induction (observed at 12 and 24 h from the end of treatment (FET)) triggered a production of ethylene (Et; +35% compared with Bc^−^/O_3_^−^ leaves), which was preceded by an increase of salicylic acid (SA; +45%). This result confirms a crosstalk between SA- and Et-related signaling pathways in lesion spread. The ozone induced an early synthesis of Et followed by jasmonic acid (JA) and SA production (about 2-fold higher), where Et and SA signaling triggered reactive oxygen species production by establishing a feedback loop, and JA attenuated this cycle by reducing Et biosynthesis. In *Bc*^+^ + O_3_^+^ leaves, Et peaked at 6 and 12 h FET, before SA confirmed a crosstalk between Et- and SA-related signaling pathways in lesion propagation. In O_3_^+^ + *Bc*^+^ leaves, the H_2_O_2_ induction triggered an accumulation of JA and Et, demonstrating a synergistic action in the regulation of defence reactions. The divergence in these profiles suggests a rather complex network of events in the transcriptional regulation of genes involved in the systemic acquired resistance.

## 1. Introduction

Grapevine (*Vitis vinifera* L.) is one of the most economically important crops worldwide, and the increases in the wine production rate demand require changes in the agricultural, processing and manufacturing practices to make them sustainable [1]. To meet this intensive demand and the qualitative and quantitative production standards, a large use of agrochemicals is often required. While vines are grown on 3.2 million hectares in the European Union (EU, equivalent to about 4% of the total cultivated areas; [2]), agrochemical applications against grapevine pathogens reach up to 60% of the total plant protection products [3]. Since their introduction, *Plasmopara viticola* Berk. and M.A. Curtis, *Erysiphe necator* Schwein, and *Botrytis cinerea* Pers. (the causal agents of downy mildew, powdery mildew and grey mould, respectively) represent the most important grapevine diseases by accounting for the largest number of treatments in vineyards worldwide [4,5]. Issues associated with agrochemicals and consumer demand for residue free products have stimulated research and the wine industry into new and eco-friendly tools for sustainable pest management and vine protection [5,6].

In the context of reducing the use of traditional chemicals, ozone (O_3_) application is one of the most promising methods that come to meet these needs. It is starting to be used as an antimicrobial agent for food treatment, storage and processing [7], and it can be considered an alternative phytosanitary treatment in the vineyard [6,8,9]. Being made up of three fairly stable oxygen atoms, O_3_ is a powerful oxidizing unit and therefore a strong disinfecting agent. Consequently, its use may have many advantages in food and wine industry, such as in control of noxious microorganisms [10,11]. The microbicidal action of is gaining attention due to the fact that no residues are present on the product after treatment (O_3_ decomposes spontaneously in water into hydrogen peroxide and hydroxide-radicals), and no aeration to remove the gas is needed [12]. When applied at an adequate and controlled concentration, O_3_ can have germicidal effects on plant pathogens by oxidizing their vital cellular components (e.g., lipids membrane, amino acids, and proteins), and thereby reducing their growth [13,14]. Ozone acts against unsaturated lipids in the microbial cell membranes causing a leakage of their contents, and eventually, microbial lysis [15]. In addition to these effects, O_3_ also induces changes in microbial cellular metabolism by inactivating enzymes such as thiokinases, acyl-CoA-thioesterase, and acyltransferases [14]. Microbial cellular redox potential is also affected as a result of modified glutathione enzyme function and activity, hence hampering microbial proliferation [15].

Ozone is usually approached as a crucial environmental stressor [16], but some investigations have also highlighted its potential role as “eustressor” [17]. Short-term exposures of plants to adequate and controlled dosages of O_3_ have been proposed as a tool to increase nutraceutical quality, since they commonly trigger antioxidants levels without compromising plant performance [8,17,18]. Plant response to O_3_ resembles the biotic defense reactions mimicking biochemical and molecular events and includes two steps: The first is a biphasic oxidative burst with a rapid, massive and transient increase in apoplastic reactive oxygen species (ROS) production; the second is the induction of pathogen-like responses, such as local programmed cell death (PCD) and hypersensitive response (HR; [19,20]). Reactive oxygen species promote an orchestrated and tightly regulated process that involves, among others, different phytohormones and/or signaling molecules such as ethylene (Et), salicylic (SA) and jasmonic (JA) acids. The similarities between plant reaction to pathogens and O_3_ therefore make the pollutant a useful non-invasive tool to elicit and study the signaling wave, which can occur as a cross response to both biotic and abiotic apoplastic-ROS-promoting stresses [21].

To the best of our knowledge, O_3_-treatments of grapevine are scarce [22], although grapevine is regarded as sensitive to O_3_ in terms of leaf visible injury [23,24]. Damage caused by O_3_ on grapevine leaves includes anatomical modifications at the mesophyll level and structural changes in the cuticle [25]. Moreover, in our previous study, we demonstrated that a single pulse of O_3_ (100 ppb for 3 h) is effective in stimulating the expression of the systemic acquired resistance (SAR)-related genes without affecting grapevine physiological status [18], by confirming that O_3_ mimics molecular events induced by pathogens [26]. Considering the above-mentioned issues, one of the main aims of the present study was that of characterizing at functional and molecular level the “indirect” protective mechanism(s) induced by O_3_ treatment (at the same dose used in the previous study) through the induction of defense responses in *V. vinifera* plants artificially inoculated with *B. cinerea* (*Bc*). In addition, another specific aim of this study was to assess whether priming effects resulting from O_3_ treatment or inoculation with *Bc* lead to protection against pathogen attack (preventive effect) or suppression of fungal inoculation (curative effect). Specifically, our goal was that of answering the following questions: (i) How much ROS are induced by *Bc* inoculation and O_3_ treatment? (ii) What hormonal mechanisms are activated in response to individual treatments (*Bc* or O_3_) and sequential double-treatment conditions (*Bc* + O_3_ or O_3_ + *Bc*)? (iii) What defence-related genes may play a pivotal role in the grapevine’s adaptive response during single- and sequential double-treatments? We postulate a protective effect of O_3_ against *Bc* inoculation and that the interactive effects of both treatments may depend on the nature of the pre-treatment (*Bc* or O_3_).

## 2. Materials and Methods

### 2.1. Biological Material and Experimental Design

**Figure 1:**
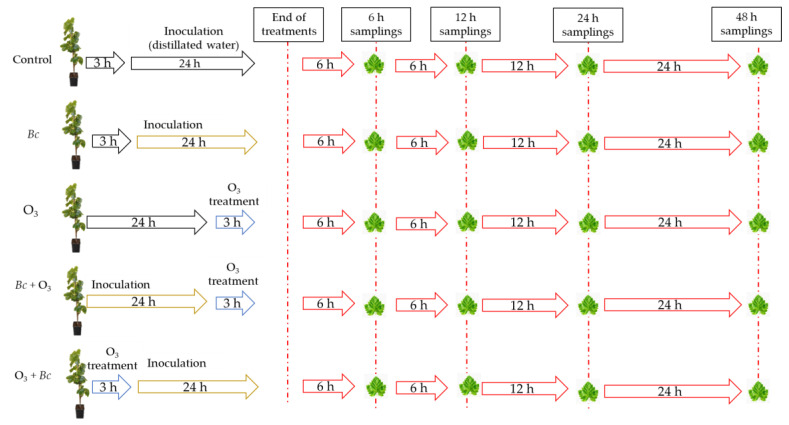
Visual summary of the whole experimental steps (inoculation with *Botrytis cinerea* (*Bc*), ozone (O_3_) treatment and their combination, and sampling timing)). Abbreviations: *Bc*^−^/O_3_^−^, uninoculated and maintained in filtered air; *Bc*^+^/O_3_^−^, inoculated with *Bc* and maintained in filtered air; *Bc*^−^/O_3_^+^, uninoculated and treated with O_3_ (100 ppb, 3 h); *Bc*^+^ + O_3_^+^, inoculated with *Bc* and then subjected to O_3_ treatment; O_3_^+^ + *Bc*^+^, treated with O_3_ and then inoculated with *Bc*.

Experimental activities were carried out at the field station of San Piero a Grado (Pisa, Italy; 43°40′48″ N, 10°20′48″ E, 2 m a.s.l.) run by the Department of Agriculture, Food and Environment (DAFE) of the University of Pisa. At the beginning of June 2021, one hundred three-year old potted plants of *V. vinifera* cv. Sangiovese grafted onto 1103 Paulsen (obtained from a local commercial nursery) were placed in a greenhouse under controlled irrigation for 1 month. In July 2021, sixty uniformly sized plants were selected and inoculated with *Bc*. The strain used for artificial inoculation (8335) was previously isolated from naturally infected *V. vinifera* leaves and preserved in the DAFE fungal collection. *Botrytis cinerea* isolate was grown on potato dextrose agar (39 g L^−1^ Sigma Aldrich, Milan, Italy) amended with streptomycin sulphate (0.1 g L^−1^, Gold Biotechnology, Saint Loius, MO, USA) in Petri dishes (Ø 9 cm) and incubated for 7 consecutive days at 23 °C and a 12-h photoperiod. Liquid cultures of *Bc* were prepared in Erlenmeyer flasks (0.5 L) containing a sterile solution of sucrose (2% *w*/*v*) and yeast extract (0.05% *w*/*v*), incubated for two days in an orbital shaker (711 CT, Asal, Milan, Italy) set at 150 rpm, and kept under room conditions. Spore concentration was determined using a Bürker hemocytometer chamber (Henneberg-Sander, Giessen Lützellinden, Germany) and initial conidia concentration was adjusted to 10^5^ spores mL^−1^. The adaxial and abaxial surfaces of *V. vinifera* leaves were sprayed with the spore suspension of *Bc* for a total of 5 mL per plant, and immediately bagged in clear plastic bags for 24 h in order to ensure a proper humidity level. Uninoculated plants were sprayed with sterile solution of sucrose (2% *w*/*v*) and yeast extract (0.05% *w*/*v*) for mock inoculation. After 48 h, plants were equally subdivided into five sets (three of which subjected to a single pulse of O_3_, 100 ppb for 3 h) and named as follow: *Bc*^−^/O_3_^−^ (uninoculated and maintained in filtered air), *Bc*^+^/O_3_^−^ (inoculated with *Bc* and maintained in filtered air), *Bc*^−^/O_3_^+^ (uninoculated and treated with O_3_), *Bc*^+^ + O_3_^+^ (inoculated with *Bc* and then subjected to O_3_ treatment), and O_3_^+^ + *Bc*^+^ (treated with O_3_ and then inoculated with *Bc*). All plants were placed in four fumigation chambers inside a greenhouse with natural lighting (the average photon flux density during measurements was around 500 μmol photons m^−2^ s^−1^ at plant height) for acclimation and kept under charcoal-filtered air (twenty-five plants in each chamber). Uninoculated plants were maintained under charcoal-filtered air at a negligible O_3_ concentration (controls, O_3_ concentration < 5 ppb) into two fumigation facilities for 27 h (*Bc*^−^/O_3_^−^). Similarly, *Bc*^+^/O_3_^−^ plants were maintained under charcoal-filtered air, and after 3 h they were inoculated with *Bc* (as previously reported). After 24 h, uninoculated and inoculated plants (*Bc*^−^/O_3_^+^ and *Bc*^+^ + O_3_^+^) were exposed to a single pulse of O_3_ (100 ppb for 3 h) into two fumigation facilities. Conversely, O_3_^+^ + *Bc*^+^ plants were exposed to a single pulse of O_3_ and after 3 h they were inoculated with *Bc* (as previously reported). Treatments and sampling times are graphically described in Figure 1. Microscopic observations were performed after 48 h from the end of the treatment (Section 2.2). At 6, 12, 24 and 48 h from the end of the (single- and double-) treatment (FET, 27 h), ten fully expanded leaves (equally distributed over plant height) were harvested from 5 randomly selected vines, immediately frozen in liquid nitrogen, and then freeze-dried and stored at −80 °C until biochemical and molecular analyses (Section 2.3 and Section 2.4).

### 2.2. Microscopic Observations

*Botrytis cinerea* infection processes were microscopically investigated by staining the hyphal structures developed in *V. vinifera* leaves. Leaf tissues have been cut in 1-cm fragments and suspended in a mixture of alcohol (95%) and lactophenol cotton blue solution (2:1), boiled for 1.5 min, and removed after 48 h. Samples have been washed with distilled water, and maintained for 30 min in chloral hydrate:water solution (2:1) according to Shipton and Brown (1962). Finally, stained leaf fragments have been fixed on glasses slides with glycerol (50%) for visualization using a transmitted light/fluorescence contrast microscope (DM 4000^®^ B led, Leica, Wetzlar, Germany). Photomicrographs were taken with a Canon PowerShot S50^®^ camera (Canon Italia, Milan, Italy).

### 2.3. Biochemical Analysis

Hydrogen peroxide (H_2_O_2_) content was measured using the AmplexTM Red Hydrogen Peroxide/Peroxidase Assay Kit (Molecular Probes, Life Technologies Corp., Carlsbad, CA, USA), according to [27]. Frozen foliage samples (50 mg) were added to 1 mL of 20 mM potassium-phosphate (K/P) buffer (pH 6.5), incubated for 30 min at 25 °C in the dark, and determined by using a Victor3 1420 Multilabel Counter microplate reader (Perkin Elmer Inc., Waltham, MA, USA) at 530 and 590 nm for the excitation and emission of resorufin fluorescence, respectively. The superoxide radical (^●^O_2_^−^) content was measured by the reduction of a tetrazolium dye sodium, 3′-(1-[phenylamino carbonyl]-3,4-tetrazolium)-bis(4-methoxy-6-nitro) benzene-sulfonic acid hydrate (XTT) by O_2_ to soluble formazan XTT according to [28]. Frozen foliage samples (30 mg) were added to 1 mL of 50 mM K/P buffer (pH 7.5), incubated for 30 min at 25 °C in the dark, and determined with the same fluorescence/absorbance microplate reader reported before at 470 nm, after subtracting the background absorbance due to the buffer solution and the assay reagents.

Ethylene (Et) emission was determined according to [29] with some modifications. Fifty minutes after leaf excision, Et production was measured by enclosing around 2.5 g of leaf samples (cut few millimeters below the petiole) in air-tight glass jars (15 mL). Gas samples (1 mL) were taken from the headspace of the jars (through a hypodermic syringe), after incubation for 2 h at room temperature. Ethylene concentrations were measured using an Agilent 8890B gas chromatograph equipped with an Agilent HP-PLOT/Q + PT capillary column (30 m × 0.32 mm; coating thickness 0.20 μm), and an Agilent 5977B single quadrupole mass detector (Agilent Technologies Inc., Santa Clara, CA, USA). Analytical conditions were as follows: The carrier gas was helium with a flow rate of 1 mL min^−1^; the injector and the transfer line were set at 170 and 180 °C, respectively. Quantification was performed against an external standard.

Salicylic acid (SA) content was determined according to [30] with some modifications. Frozen foliage samples (120 mg) were added to 1 mL 90% (*v*/*v*) methanol (MeOH), vortexed and sonicated for 10 min. After centrifugation at 10,000× *g* for 15 min at room temperature, the supernatant was transferred, and the pellet was re-extracted in 0.5 mL 100% MeOH following the same procedure. Supernatants from both extractions were combined and evaporated at 35 °C under a vacuum (RVC 2-25 CDplus, Martin Christ Gefriertrocknungsanlagen GmbH, Osterode am Harz, Germany). The residue was resuspended in 0.25 mL of 5% (*w*/*v*) trichloroacetic acid and partitioned twice using 0.8 mL of a 1:1 (*v*/*v*) mixture of ethyl acetate/cyclohexane. The upper phase containing free SA was concentrated at 35 °C under a vacuum, and the lower aqueous phase (with conjugated SA) was hydrolyzed by adding 0.3 mL 8 N HCl and incubating for 60 min at 80 °C. The SA collected from both the upper and lower phases were combined and dissolved in 500 µL of the mobile phase, containing 0.2 M sodium acetate buffer (pH 5.5), water (90%) and MeOH (10%). The separation was performed at 40 °C by an ultra-high pressure liquid chromatography (UHPLC) Dionex UltiMate 3000 system equipped with an Acclaim 120 C18 column (5 μm particle size, 4.6 mm internal diameter × 150 mm length), and an UltiMate™ 3000 Fluorescence Detector (Thermo Scientific, Waltham, MA, USA) with excitation at 305 nm and emission at 407 nm. The flow rate was 0.8 mL minute^−1^. To quantify the SA content, known amounts of pure standard (0.1–100 ng mL^−1^) were injected into the UHPLC system and an equation, correlating the peak area to SA concentration, was formulated. Endogenous total SA (conjugated and free forms) content has been reported.

Jasmonic acid (JA) was determined according to [29] with minor modifications. Frozen foliage samples (100 mg) were added to 1 mL of MeOH, sonicated three times for 10 min and centrifuged at 13,000× *g* for 30 min at room temperature. The supernatants were filtered and evaporated at 37 °C under a vacuum for 10 min. The residue was re-suspended with 750 μL of ethyl acetate. The extract was injected into a GC-MS (as previously reported) equipped with an Agilent DB-5MS (UI) capillary column (30 m × 0.25 mm; coating thickness 0.25 μm). Analytical conditions were as follows: the carrier gas was helium with a flow rate of 1 mL minute^−1^; the injector and the transfer line were set at 280 and 340 °C, respectively. The temperature program was as follows: the initial column temperature was set at 70 °C for 4 min, increasing to 300 °C at 10 °C minute^−1^ for 2 min, and then increasing to 340 °C at 5 °C minute^−1^, holding until the end of the analysis. Source and quadrupole temperatures were set at 230 and 150 °C, respectively. The mass data were collected in the electron impact mode at 70 eV with a scan range of 40–500 *m*/*z*, and the quantification was performed at the selected-ion monitoring mode at *m*/*z* 151 amu by using MassHunter Workstation (version 10.0, Agilent Technologies Inc., Santa Clara, CA, USA).

### 2.4. Molecular Analysis

For each sampling time, the expression of resistance-related genes was determined. As SAR marker genes, genes encoding pathogenesis-related proteins 1 and 6 (*PR1* and *PR6*, respectively), chitinases B and IV (*CHIT B* and *CHIT IV*, respectively), glutathione S-transferase (*GST*) and *β-1,3 glucanase* were selected (Heath, 2007). One hundred mg of ground tissue were used for RNA extraction, using Spectrum™ Plant Total RNA Kit (Sigma-Aldrich, Milan, Italy), including DNA digestion with On-Column DNase I Digestion Set (Sigma-Aldrich, Milan, Italy). RNA concentration and purity were determined with Nanodrop 2000 spectrophotometer (Thermo Scientific, Milan, Italy). The integrity of the extracted RNA was checked on a 1% (*w*/*v*) agarose gel. Reverse transcription to cDNA was performed using a 50 ng RNA template and 4 μL of ReadyScript™ cDNA Synthesis Mix (Sigma-Aldrich, Milan, Italy) in a final volume of 20 μL. The PCR conditions were set according to the manufacturer’s protocol. Gene-specific primers and dilutions were the same used by [18]. Sample analyses was performed using the SYBR Green PCR Master Mix (Life Technologies™, Milan, Italy), with a final reaction volume of 10 μL, running on the CFX Connect RT-qPCR System (BioRad Laboratories, Inc., Hercules, CA, USA). The RT-qPCR cycle was set as follows: initial denaturation at 95 °C for 2 min, followed by 40 cycles of amplification with denaturation at 95 °C for 15 s, and annealing and elongation at 60 °C for 1 min. After the 40 cycles, a melt cycle was performed at 95 °C for 15 s, 60 °C for 1 min, 95 °C for 15 s and 60 °C for 15 s. A negative control was performed in all qPCRs runs. For data analysis, the comparative Ct (2^−ΔΔCt^) method described in [31] was used. Expression levels were normalised using the ubiquitin (*VvUBC*) housekeeping gene. The relative quantification of each gene tested was calculated using the 2^−ΔΔCt^ method, taking as reference the control of each sampling time. The forward and reverse sequences, GenBank Accession, as well as the primer efficiencies, are given in Supplementary Table S1.

### 2.5. Statistical Analysis

For all the experiments, the robustness of data among replicates was verified according to the results of the Shapiro-Wilk for normality and Levene tests for the homogeneity of variance. Data were submitted to analysis of variance (ANOVA) and comparisons among means were determined by the Tukey’s HSD post-hoc test by using JMP Pro 14 software (SAS Institute Inc., Cary, NC, USA) in order to evaluate the effect of the treatments (*Bc*^−^/O_3_^−^ vs. *Bc*^+^/O_3_^−^, *Bc*^−^/O_3_^−^ vs. *Bc*^−^/O_3_^+^, *Bc*^−^/O_3_^−^ vs. *Bc*^+^ + O_3_^+^, *Bc*^−^/O_3_^−^ vs. O_3_^+^ + *Bc*^+^), time (6, 12, 24, and 48 h), and their combination. For all the analyses, *p* ≤ 0.05 was assumed as a significant level. Gene expression data were compared by one-way ANOVA and Tukey’s HSD post-hoc test at *p* < 0.05 using GraphPad Prism 7.01 (GraphPad Software, La Jolla, CA, USA) separately for the different sampling times.

## 3. Results

### 3.1. Macroscopic and Microscopic Symptoms

**Figure 2:**
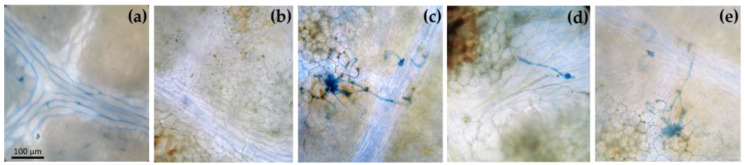
Leaves of *Vitis vinifera* var. Sangiovese coloured with lactophenol-cotton blue and observed in an optical after 48 h from the end of each treatment. From left to right: Leaves from plants (**a**) uninoculated and maintained in filtered air (*Bc*^−^/O_3_^−^), (**b**) uninoculated with *Botrytis cinerea* (*Bc*) and treated with ozone (O_3_, 100 ppb, 3 h; *Bc*^−^/O_3_^+^), (**c**) inoculated with *B. cinerea* and maintained in filtered air (*Bc*^+^/O_3_^−^), (**d**) inoculated with *B. cinerea* and then subjected to O_3_ treatment (*Bc*^+^ + O_3_^+^), and (**e**) treated with O_3_ and then inoculated with *B. cinerea* (O_3_^+^ + *Bc* ^+^).

Microscopic observations allowed for a first evaluation of the effective penetration of *Bc* in different areas of the leaves (Figure 2). Uninoculated leaves (*Bc*^−^/O_3_^−^ and *Bc*^−^/O_3_^+^) did not show the presence of stained fungal structures (Figure 2a,b). Conversely, germ tubes emerged from conidia, elongated and their hyphae spread throughout leaf tissues starting from 48 h after inoculation (Figure 2c). Similar structures were found on *Bc*^+^ + O_3_^+^ and O_3_^+^ + *Bc*^+^ leaves (Figure 2d,e). In both cases, germ tubes did not elongate well, and their hyphae were slightly spread over the leaves. At the end of O_3_ exposure, leaves were macroscopically symptomless.

### 3.2. Biochemical Responses

**Figure 3:**
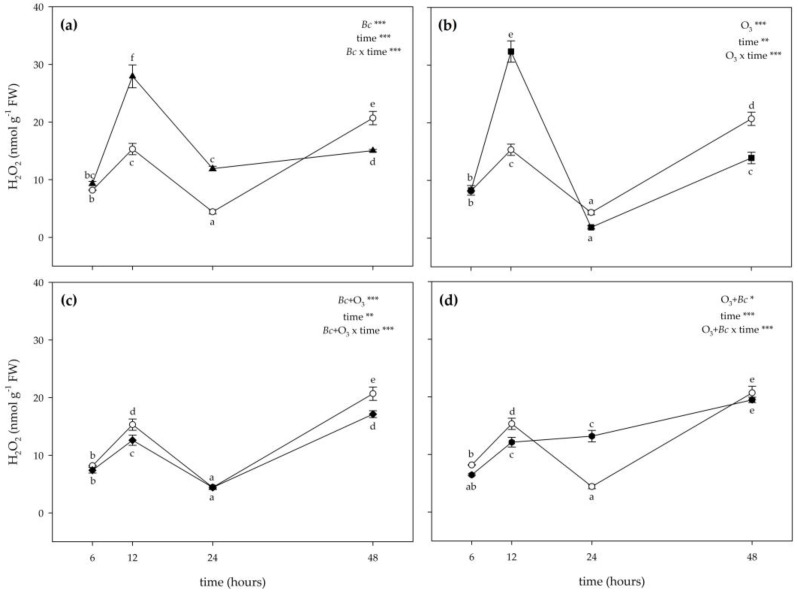
Time course of hydrogen peroxide (H_2_O_2_) content in leaves of *Vitis vinifera* var. Sangiovese uninoculated and maintained in filtered air (*Bc*^−^/O_3_^−^; empty circle) compared to samples (**a**) inoculated with *Botrytis cinerea* (*Bc*) and maintained in filtered air (*Bc*^+^/O_3_^−^; filled triangle), (**b**) uninoculated with *B. cinerea* and treated with ozone (O_3_, 100 ppb, 3 h; *Bc*^−^/O_3_^+^; filled square), (**c**) inoculated with *B. cinerea* and then subjected to O_3_ treatment (*Bc^+^* + O_3_^+^; filled diamond), and (**d**) treated with O_3_ and then inoculated with *B. cinerea* (O_3_^+^ + *Bc^+^*; filled circle). Data are shown as mean ± standard deviation. The measurements are carried out at 6, 12, 24 and 48 h from the end of the treatment. In each graph, the results of two-way ANOVA are reported, asterisks showing the significance of factors /interaction for: *** *p* ≤ 0.001; ** *p* ≤ 0.01; * *p* ≤ 0.05. According to Tukey’s HSD post hoc test, different letters indicate significant differences (*p* ≤ 0.05). Abbreviation: FW, fresh weight.

The two-way ANOVA of H_2_O_2_ content showed that the effects of treatments, time and their combination were significant (Figure 3). The inoculation with *Bc* significantly stimulated the production of H_2_O_2_ at 12 and 24 h FET (about 2- and 3-fold higher than controls; Figure 3a). Conversely, a slight reduction of H_2_O_2_ content was observed in *Bc*^+^/O_3_^−^ leaves at 48 h FET (−28%). A variable O_3_ effect was instead reported on H_2_O_2_ levels: they increased at 12 h FET (2-fold higher than controls), did not show differences at 24 h FET, and decreased at 48 h FET (−33%; Figure 3b). In *Bc*^+^ + O_3_^+^ leaves, a slight reduction of H_2_O_2_ levels was observed at 12 and 48 h FET (−18 and −17%, respectively). No significant effects were reported at 24 h FET (Figure 3c). In O_3_^+^ + *Bc*^+^ leaves, the concentration of H_2_O_2_ did not show a clear trend: it was lower than controls at 12 h FET (−21%), increasing at 24 h FET (3-fold higher than untreated material), and showing no differences at 48 h FET (Figure 3d). No significant effects were reported at 6 h FET for this parameter independently of the treatment (Figure 3).

**Figure 4:**
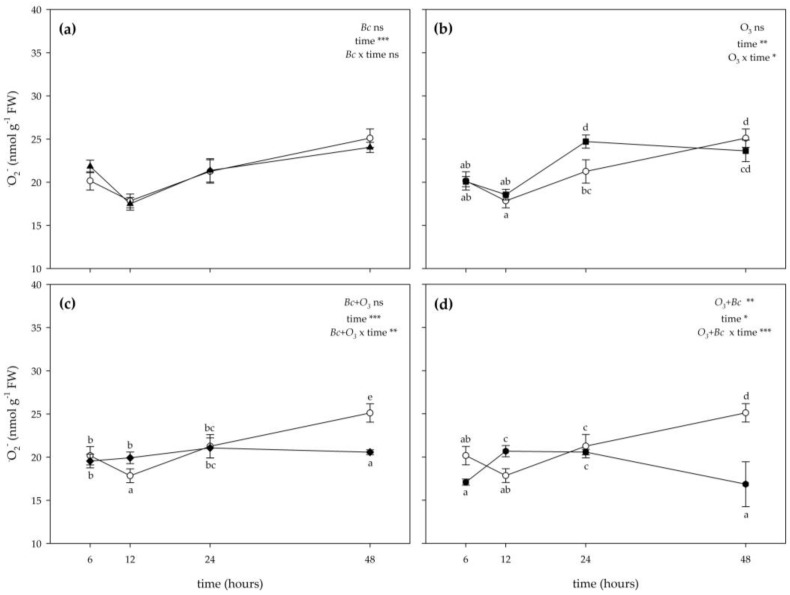
Time course of superoxide radical (^●^O_2_^−^) generating rate in leaves of *Vitis vinifera* var. Sangiovese uninoculated and maintained in filtered air (*Bc*^−^/O_3_^−^; empty circle) compared to samples (**a**) inoculated with *Botrytis cinerea* (*Bc*) and maintained in filtered air (*Bc*^+^/O_3_^−^; filled triangle), (**b**) uninoculated with *B. cinerea* and treated with ozone (O_3_, 100 ppb, 3 h; *Bc*^−^/O_3_^+^; filled square), (**c**) inoculated with *B. cinerea* and then subjected to O_3_ treatment (*Bc*^+^ + O_3_^+^; filled diamond), and (**d**) treated with O_3_ and then inoculated with *B. cinerea* (O_3_^+^ + *Bc*^+^; filled circle). Data are shown as mean ± standard deviation. The measurements are carried out at 6, 12, 24 and 48 h from the end of the treatment. In each graph, results of two-way ANOVA are reported, asterisks showing significance of factors/interaction for: *** *p* ≤ 0.001; ** *p* ≤ 0.01; * *p* ≤ 0.05; ns *p* > 0.05. According to Tukey’s HSD post hoc test, different letters indicate significant differences (*p* ≤ 0.05). Abbreviation: FW, fresh weight.

The two-way ANOVA of •O_2_^−^ content showed that the effects of treatments (only O_3_ fumigation and then inoculation with *Bc* (O_3_^+^ + *Bc*^+^)), time and their combination (except in the case of “inoculation with *B. cinerea* (*Bc*^+^/O_3_^−^) × time”; Figure 4a)) were significant (Figure 4). Ozone slightly stimulated the production of •O_2_^−^ only at 24 h FET (+16% compared with controls; Figure 4b). However, no significant effects were reported at other times of the analysis. In leaves of *V. vinifera* subjected to both combined treatments (*Bc*^+^ + O_3_^+^ and O_3_^+^ + *Bc*^+^), the concentration of •O_2_^−^ did not show a clear trend; it was higher than control at 12 h FET (+12 and +16%, in *Bc*^+^ + O_3_^+^ and O_3_^+^ + *Bc*^+^ leaves, respectively), showing no differences at 24 h FET, and decreasing at 48 h FET (−18 and −33%, respectively; Figure 4c,d). No significant effects were reported at 6 h FET for this parameter independently of the treatment (Figure 4).

**Figure 5:**
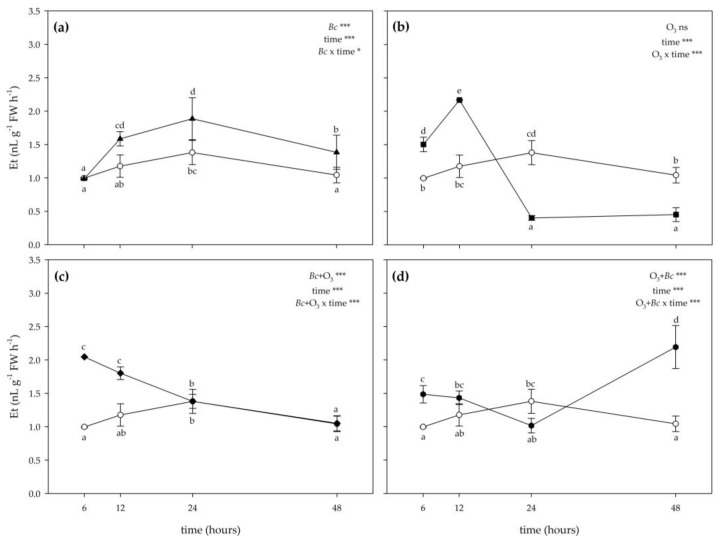
Time course of ethylene (Et) emission in leaves of *Vitis vinifera* var. Sangiovese uninoculated and maintained in filtered air (*Bc*^−^/O_3_^−^; empty circle) compared to (**a**) inoculated with *Botrytis cinerea* (*Bc*) and maintained in filtered air (*Bc*^+^/O_3_^−^; filled triangle), (**b**) uninoculated with *B. cinerea* and treated with ozone (O_3_, 100 ppb, 3 h; *Bc*^−^/O_3_^+^; filled square), (**c**) inoculated with *B. cinerea* and then subjected to O_3_ treatment (*Bc*^+^ + O_3_^+^; filled diamond), and (**d**) treated with O_3_ and then inoculated with *B. cinerea* (O_3_^+^ + *Bc*^+^; filled circle). Data are shown as mean ± standard deviation. The measurements are carried out at 6, 12, 24 and 48 h from the end of the treatment. In each graph, results of the two-way ANOVA are reported, asterisks showing significance of factors/interaction for: *** *p* ≤ 0.001; * *p* ≤ 0.05; ns *p* > 0.05. According to Tukey’s HSD post hoc test, different letters indicate significant differences (*p* ≤ 0.05). Abbreviation: FW, fresh weight.

The two-way ANOVA of Et levels showed that the effects of treatments (except in the case of O_3_ fumigation (*Bc*^−^/O_3_^+^)), time and their combination were significant (Figure 5). The inoculation with *Bc* significantly stimulated the production of Et at 12, 24 and 48 h FET (+35, +37 and +33% compared with controls, respectively; Figure 5a). Conversely, no significant effects were reported at 6 h FET. A variable O_3_ effect was instead reported on Et values: they increased at 6 and 12 h FET (+51 and +84%, respectively), and decreased at 24 and 48 h FET (−71 and −57%; Figure 5b). In *Bc*^+^ + O_3_^+^ leaves, a marked increase of Et levels was observed at 6 and 12 h FET (about 2-fold higher than controls). No significant effects were reported at the following times of analysis (Figure 5c). In O_3_^+^ + *Bc*^+^ leaves, the emission of Et did not show a clear trend; it was higher than controls at 6 h FET (+49%), showing no differences at 12 and 24 h FET, and increasing again at 48 h FET (2-fold higher than untreated samples; Figure 5d).

**Figure 6:**
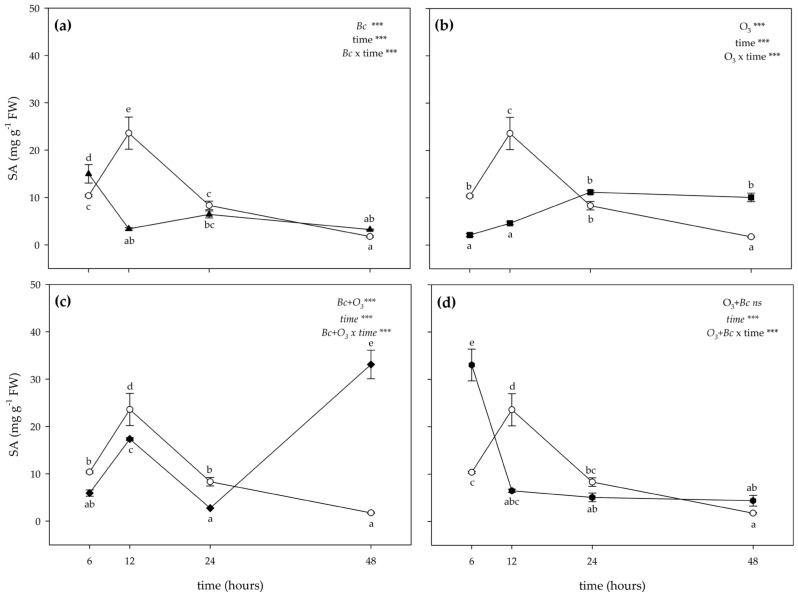
Time course of salicylic acid (SA) content in leaves of *Vitis vinifera* var. Sangiovese uninoculated and maintained in filtered air (*Bc*^−^/O_3_^−^; empty circle) compared to (**a**) inoculated with *Botrytis cinerea* (*Bc*) and maintained in filtered air (*Bc*^+^/O_3_^−^; filled triangle), (**b**) uninoculated with *B. cinerea* and treated with ozone (O_3_, 100 ppb, 3 h; *Bc*^−^/O_3_^+^; filled square), (**c**) inoculated with *B. cinerea* and then subjected to O_3_ treatment (*Bc*^+^ + O_3_^+^; filled diamond), and (**d**) treated with O_3_ and then inoculated with *B. cinerea* (O_3_^+^ + *Bc*^+^; filled circle). Data are shown as mean ± standard deviation. The measurements are carried out at 6, 12, 24 and 48 h from the end of the treatment. In each graph, the results of two-way ANOVA are reported, asterisks showing significance of factors/interaction for: *** *p* ≤ 0.001; ns *p* > 0.05. According to Tukey’s HSD post hoc test, different letters indicate significant differences (*p* ≤ 0.05). Abbreviation: FW, fresh weight.

The two-way ANOVA of SA content showed that the effects of treatments (except in the case of O_3_ fumigation and then inoculation with *B. cinerea* (O_3_^+^ + *Bc*^+^)), time and their combination were significant (Figure 6). A variable effect of inoculation with *Bc* was instead reported on SA levels; they were increased at 6 h FET (+45% compared with controls), decreased at 12 FET (−83%), and did not show differences at 24 and 48 h FET (Figure 6a). In *Bc*^−^/O_3_^+^ leaves, the content of SA did not show a clear trend: it was lower than controls at 6 and 12 h FET (−79 and −77% compared with controls respectively), showing no differences at 24 h FET, and increased at 48 h FET (+34%; Figure 6b). In *Bc*^+^ + O_3_^+^ leaves, a significant reduction of SA levels was observed at 12 and 24 h FET (−14 and −69%, respectively). Conversely, an accumulation of SA was observed at 48 h FET (more than 15-fold higher than controls). No significant effects were reported at 6 h FET (Figure 6c). In O_3_^+^ + *Bc*^+^ leaves, the concentration of SA did not show a clear trend; it was higher than that of controls at 6 h FET (about 4-fold higher than untreated material), decreasing at 12 h FET (−67%), and showing no differences at 24 and 48 h FET (Figure 6d).

**Figure 7:**
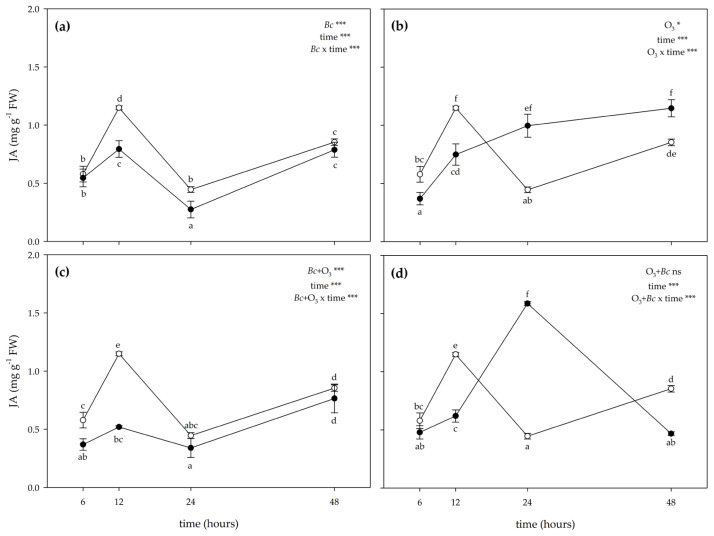
Time course of jasmonic acid (JA) content in leaves of *Vitis vinifera* var. Sangiovese uninoculated and maintained in filtered air (*Bc*^−^/O_3_^−^; empty circle) compared to (**a**) inoculated with *Botrytis cinerea* (*Bc*) and maintained in filtered air (*Bc*^+^/O_3_^−^; filled triangle), (**b**) uninoculated with *B. cinerea* and treated with ozone (O_3_, 100 ppb, 3 h; *Bc*^−^/O_3_^+^; filled square), (**c**) inoculated with *B. cinerea* and then subjected to O_3_ treatment (*Bc*^+^ + O_3_^+^; filled diamond), and (**d**) treated with O_3_ and then inoculated with *B. cinerea* (O_3_^+^ + *Bc*^+^; filled circle). Data are shown as mean ± standard deviation. The measurements are carried out at 6, 12, 24 and 48 h from the end of the treatment. In each graph, results of two-way ANOVA are reported, asterisks showing significance of factors/interaction for: *** *p* ≤ 0.001; * *p* ≤ 0.05; ns *p* > 0.05. According to Tukey’s HSD post hoc test, different letters indicate significant differences (*p* ≤ 0.05). Abbreviation: FW, fresh weight.

The two-way ANOVA of JA content showed that the effects of treatments, time and their combination were significant (Figure 7). The inoculation with *Bc* significantly decreased the concentration of JA at 12 and 24 h FET (−31% and −38% compared with controls, respectively; Figure 7a). No significant effects were reported at 6 and 48 h FET. In *Bc*^−^/O_3_^+^ leaves, O_3_ treatment significantly decreased JA levels at 6 and 12 h FET (about 2-fold lower than controls; Figure 7b). Conversely, an accumulation of JA was observed at 24 and 48 h FET (about 2-fold higher than controls). In *Bc*^+^ + O_3_^+^ leaves, a significant reduction of JA content was observed at 6 and 12 h FET (about 2-fold lower than controls, Figure 7c). No significant effects were reported at 24 and 48 h FET. In O_3_^+^ + *Bc*^+^ leaves, a significant reduction of JA values was observed only at 12 and 48 h FET (−46 and −45%, respectively; Figure 7d). Conversely, an accumulation of JA was observed at 24 h FET (more than 3-fold higher than controls). No significant effects were reported at 6 h FET.

**Figure 8:**
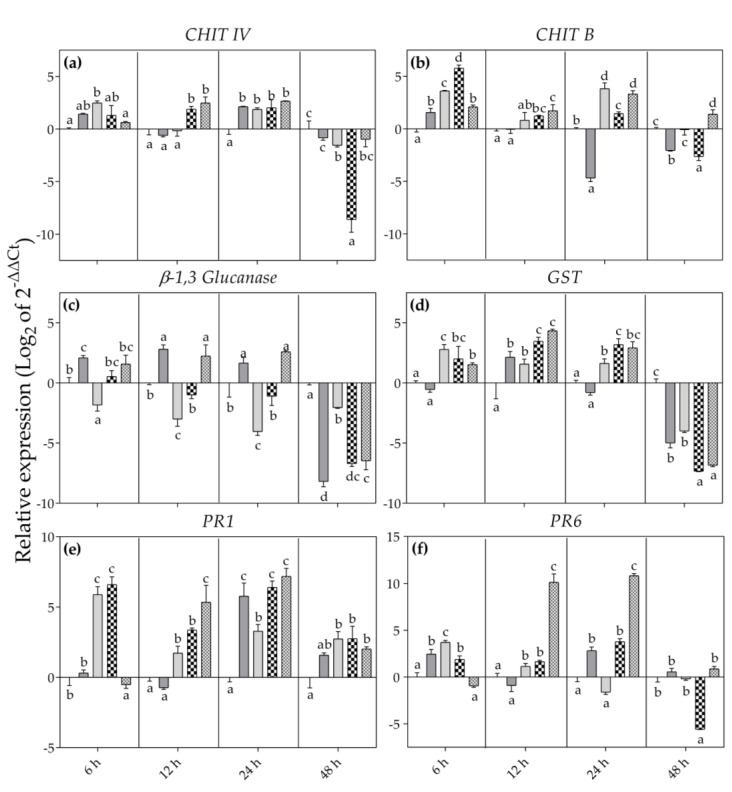
Relative expression level of *CHIT IV* (**a**), *CHIT B* (**b**), *β-1,3 Glucanase* (**c**), *GST* (**d**), *PR1* (**e**) and *PR6* (**f**) analysed by RT-qPCR in leaves of *Vitis vinifera* var. Sangiovese uninoculated and maintained in filtered air (*Bc*^−^/O_3_^−^; black) and (**a**) inoculated with *Botrytis cinerea* (*Bc*) and maintained in filtered air (*Bc*^+^/O_3_^−^; dark grey), (**b**) uninoculated with *B. cinerea* and treated with ozone (O_3_, 100 ppb, 3 h; *Bc*^−^/O_3_^+^; grey), (**c**) inoculated with *B. cinerea* and then subjected to O_3_ treatment (*Bc*^+^ + O_3_^+^; dark check pattern), and (**d**) treated with O_3_ and then inoculated with *B. cinerea* (O_3_^+^ + *Bc*^+^; grey check pattern). The measurements are carried out at 6, 12, 24 and 48 h from the end of the treatment. The average value of the three biological replicates is reported with bars representing standard deviation. In each sampling time, different letters indicate differences among sample values (*p* < 0.05) based on one-way ANOVA.

As far as expression data of selected genes is concerned, at 6 h FET *CHIT IV* was statistically up-regulated in *Bc*^−^/O_3_^+^ leaves (more than 2-fold higher than *Bc*^−^/O_3_^−^ ones, *p* < 0.001; Figure 8a). The other treatments showed expression levels similar to that observed in *Bc*^−^/O_3_^−^ leaves. At the same sampling time, *CHIT B* was up regulated in all the applied treatments, by reaching the highest expression in *Bc*^+^ + O_3_^+^ leaves (more than 5-fold higher than *Bc*^−^/O_3_^+^ samples, *p* < 0.001; Figure 8b). Conversely, *β-1,3 glucanase* was statistically up-regulated only in *Bc*^+^/O_3_^−^ leaves (more than 2-fold higher than *Bc*^−^/O_3_^−^ samples, *p* < 0.01), and down-regulated in *Bc*^−^/O_3_^+^ treated leaves (more than 2-fold lower than *Bc*^−^/O_3_^−^, *p* < 0.05; Figure 8c). The other treatments showed expression level like that observed in *Bc*^−^/O_3_^−^ plants. *Glutathione S-transferase* was up-regulated in all the applied treatments (except in the case of *Bc*^+^/O_3_^−^ leaves; Figure 8d). In *Bc*^−^/O_3_^+^ and *Bc*^+^ + O_3_^+^ leaves, *PR1* showed a strong up regulation (Figure 8e). Lastly, *PR6* was statistically over expressed in all the applied treatments (except in the case of O_3_^+^ + *Bc*^+^ leaves; Figure 8f).

At 12 h FET, *CHIT IV* and *CHIT B* were up regulated in *Bc*^+^ + O_3_^+^ and O_3_^+^ + *Bc*^+^ leaves (more than 2-fold higher than *Bc*^−^/O_3_^−^, *p* < 0.01 and *p* < 0.001; Figure 8a,b). At the same sampling time, the expression of *β-1,3 glucanase* was stimulated in *Bc*^+^/O_3_^−^ and O_3_^+^ + *Bc*^+^ leaves (more than 2-fold higher than *Bc*^−^/O_3_^−^, *p* < 0.001; Figure 8c). Conversely, the expression of this gene slightly declined in *Bc*^−^/O_3_^+^ leaves (more than 2-fold higher than *Bc*^−^/O_3_^−^, *p* < 0.001). *Glutathione S-transferase* was up regulated in all the applied treatments, by reaching the highest values in *Bc*^+^ + O_3_^+^ and O_3_^+^ + *Bc*^+^ leaves (more than 3- and 4-fold higher than *Bc*^−^/O_3_^−^ ones, *p* < 0.001; Figure 8d). Lastly, *PR1* and *PR6* were statistically over expressed in *Bc*^−^/O_3_^+^, *Bc*^+^ + O_3_^+^ and O_3_^+^ + *Bc*^+^ leaves, reaching the maximum values in O_3_^+^ + *Bc*^+^ samples (more than 6- and 9-fold higher than *Bc*^−^/O_3_^−^, *p* < 0.001; Figure 8e,f).

At 24 h FET, the expression level of *CHIT IV* increased in all the applied conditions (Figure 8a). Likewise, *CHIT B* was up-regulated in all treatments, with the exception of *Bc*^+^/O_3_^−^ leaves (Figure 8b). At the same sampling time, the expression of *β-1,3 glucanase* was stimulated in *Bc*^+^/O_3_^−^ and O_3_^+^ + *Bc*^+^ leaves (more than 2-fold higher than *Bc*^−^/O_3_^−^, *p* < 0.001; Figure 8c). Conversely, the expression of this gene declined in *Bc*^−^/O_3_^+^ leaves (more than 4-fold lower than *Bc*^−^/O_3_^−^, *p* < 0.001). *Glutathione S-transferase* was up regulated in all the applied treatments, with the exception of *Bc*^+^/O_3_^−^ leaves (Figure 8d), by reaching the highest values in *Bc*^+^ + O_3_^+^ leaves (more than 3-fold lower than *Bc*^−^/O_3_^−^, *p* < 0.001). With the exception of *Bc*^−^/O_3_^+^ leaves, *PR1* and *PR6* were up-regulated in all the applied treatments (Figure 8e,f).

At 48 h FET, *CHIT IV*, *CHIT B*, *β-1,3 glucanase* and *GST* were down regulated in all the applied treatments, except in the case of *Bc*^+^/O_3_^−^ and O_3_^+^ + *Bc*^+^ leaves for *CHIT IV* (Figure 8a), *Bc*^−^/O_3_^+^ leaves for *CHIT B* (Figure 8b). Conversely, *PR1* was significantly over expressed in *Bc*^−^/O_3_^+^, *Bc*^+^ + O_3_^+^ and O_3_^+^ + *Bc*^+^ leaves (Figure 8d). Lastly, *PR6* was significantly down regulated in *Bc*^+^ + O_3_^+^ leaves (more than 6-fold lower than *Bc*^−^/O_3_^−^, *p* < 0.001; Figure 8f).

## 4. Discussion

Under natural conditions, plants encounter several stress factors that occurred singularly, simultaneously or sequentially. To counteract them, plants make use of constitutive and induced defences to ensure their survival [32]. Great efforts have been made to understand the dual role of ROS in plant biology. Reactive oxygen species are required for several important signaling reactions, but they are also toxic by-products of aerobic metabolism [33]. This dual role is mainly dependent on (i) their concentration, (ii) duration and site of their action, (iii) occurrence of previous stress events, and (iv) concurrence of other constrained conditions. At high doses, ROS pose a significant threat that may eventually lead to HR-like PCD, while at low concentrations they are employed as signals that mediate at least part of the responses towards stress. However, the rapid generation of ROS represents a common plant response to different biotic and abiotic stresses, and thus a basis to unify signalling events [34].

In light of the above considerations, the first question we wanted to address was “How much ROS are induced by *Bc* inoculation and O_3_ treatment?”. It is known that *Bc* can produce ROS *in planta* (presumably via NADPH oxidases and superoxide dismutases). During the infection process, an accumulation of •O_2_^−^ was usually identified in fungal hyphal tips, whereas H_2_O_2_ was generated in the plant plasma membrane and in/around the penetrated cell wall [35]. ROS-induced processes are especially complex in the plant-*Bc* interaction. In our work, an accumulation of H_2_O_2_ was observed in *Bc*^+^/O_3_^−^ leaves at 12 and 24 h FET indicating that this ROS might be produced by *Bc* as a virulence factor [35], as confirmed by the initiation of infection process starting from 12 h FET. In particular, the increased H_2_O_2_ levels not only affect *Bc*^+^/O_3_^−^ leaves by promoting HR but might participate in *Bc* development by influencing its redox status (as confirmed by the slight reduction of H_2_O_2_ content observed at 48 h FET [36]. No significant changes were observed in terms of •O_2_^−^ throughout the whole period of the experiment. Assuming that O_3_ induces a self-propagating, active and endogenous ROS generation in the apoplast and a subsequent cellular oxidative burst, some authors have proposed that a single pulse of O_3_ can mimic pathogen infection process [16]. In this work, an accumulation of H_2_O_2_ and •O_2_^−^ was observed in *Bc*^−^/O_3_^+^ leaves during the recovery (at 12 and 24 h FET respectively), suggesting that O_3_ *per se* did not generate the H_2_O_2_ and/or •O_2_^−^, but rather has triggered a stress-related H_2_O_2_/•O_2_^−^ formation-like pathogen attack. In particular, the production peaks of these ROS in *Bc*^−^/O_3_^+^ leaves could be entirely ascribable to the plant metabolism (e.g., a transient oxidative burst; [37]. These results suggest that *Bc* infection and O_3_ treatment share many similarities during the early stages after stress initiation including ROS production and HR-like PCD activation [38]. It is worth noting that a divergence in ROS profiles and in the magnitude of their relative peaks was observed among leaves subjected to individual (*Bc*^+^/O_3_^−^ and *Bc*^−^/O_3_^+^) and sequential double-treatments (*Bc*^+^ + O_3_^+^ and O_3_^+^ + *Bc*^+^). In *Bc*^+^ + O_3_^+^ and O_3_^+^ + *Bc*^+^ leaves, no differences were observed in •O_2_^−^ extent dynamics in relation to the sequence of treatments (e.g., accumulation of •O_2_^−^ at 12 h FET). This result indicates that a transient oxidative burst occurred (also confirmed by the increased H_2_O_2_ levels observed in O_3_^+^ + *Bc*^+^ leaves at 24 h FET) by triggering an active (programmed) cell death in the host that could facilitate *Bc* to access nutrients and survive [36]. Consequently, there are different kinds of ROS that, however, might have different effects on the growth of *Bc*, as confirmed by the reduced disease progression (in terms of germ tube development and hyphae elongation) observed in *Bc*^+^ + O_3_^+^ and O_3_^+^ + *Bc*^+^ leaves. This result suggests a rather complex network of events in signal transduction, involving other molecules (e.g., phytohormones) and processes [39].

The second question was “What hormonal mechanisms are activated in response to individual treatments (*Bc* and O_3_) and sequential double treatment conditions?”. Phytohormones and signaling molecules (such as Et, SA and JA) play crucial roles in plant defences. Ethylene and JA have been connected to defences against necrotrophic pathogens (such as *Bc*), whereas SA is important in defences against biotrophic pathogens, although it also plays some role in the defence against *Bc* [40]. In *Bc*^+^/O_3_^−^ leaves, the H_2_O_2_ induction (observed at 12 and 24 h FET) triggered the synthesis of Et starting from 12 h FET, which was preceded by a marked increase of SA at 6 h FET. This result confirms a crosstalk between SA- and Et-related signaling pathways in lesion spread and propagation to surrounding cells after *Bc* infection [41]. The absence of any enhancement of JA throughout the whole period of the experiment confirms that this compound was not involved in the regulation of PCD strategies or signaling responses to *Bc* [42]. A different chronological order of the first peaks of the phytohormones/signaling molecules responsible for the initiation, propagation and containment phases was observed in *Bc*^−^/O_3_^+^ leaves. A single pulse of O_3_ induced an early synthesis of Et (at 6 and 12 h FET) followed by the production of JA and SA (at 24 (only JA) and 48 h FET), where (i) Et and SA signaling triggered ROS production (e.g., accumulation of H_2_O_2_ and •O_2_^−^ at 12 and 24 h FET, respectively) by establishing a feedback loop, and (ii) JA attenuated this cycle by reducing the ROS production, and consequently Et biosynthesis. These outcomes confirm a spatial and functional correlation between the accumulation of these phytohormones/signaling molecules and ROS in the regulation of defense reactions against O_3_ [21,37]. A different and specific crosstalk among phytohormones and signaling molecules was observed in leaves subjected to sequential double-treatments (*Bc*^+^ + O_3_^+^ and O_3_^+^ + *Bc*^+^). In *Bc*^+^ + O_3_^+^ leaves, Et peaked at 6 and 12 h FET, before SA (at 48 h FET) possibly indicating a crosstalk between Et- and SA-related signaling pathways in lesion spread and propagation to surrounding cells after *Bc* infection and O_3_ treatment. The absence of any enhancement of JA throughout the whole period of the experiment (as previously reported in *Bc*^+^/O_3_^−^ leaves) confirms that this compound was not involved in the regulation of PCD strategies or signalling responses [42]. In O_3_^+^ + *Bc*^+^ leaves, a marked production of Et and SA was observed at 6 h FET preceding that of ROS (as previously reported in *Bc*^−^/O_3_^+^ and *Bc*^+^/O_3_^−^ leaves, respectively). This result indicates that Et and SA accumulation might also be involved in the increased generation of ROS during the early stages after sequential double-stress initiation. The •O_2_^−^ and H_2_O_2_ induction (observed at 12 and 24 h FET, respectively), in turn, triggered an accumulation of JA and Et at 24 and 48 h FET, respectively, demonstrating a synergistic action in the (i) regulation of defence reactions, and (ii) activation of HR, as confirmed by the observed inhibition of mycelial growth from germinated conidia [43]. The observed divergence in phytohormones/signaling molecules profiles and in the magnitude of their relative peaks among leaves subjected to individual and sequential double-treatments suggests a rather complex network of events in transcriptional regulation, involving hormone-responsive marker genes, resistance-related genes and/or genes related to specific plant processes [39].

Finally, the third question was “What defence-related genes play a pivotal role in grapevine adaptive response during single- and sequential double-treatments?”. Plants have developed complex responses at molecular levels to increase their tolerance and to adapt to unfavourable environmental conditions. Many SAR-related genes take part in SAR activation involving two different mechanisms: the recognition of virulence products, or direct interaction with the pathogen’s biological structure [41]. The genes acting via the first mechanism are generally involved in quick and local response. Conversely, those that directly interact with the pathogens are involved in the systemic response, and their activity lasts longer [44]. In *Bc*^+^/O_3_^−^ leaves, the expression of some genes is rapidly stimulated within a few hours after *Bc* infection (e.g., *CHIT B*, *β-1,3 glucanase* and *PR6* at 6 h FET; *β-1,3 glucanase* and *GST* at 12 h FET) indicating that they are more involved in the early defence response [45]. A transient and limited over-regulation of *CHIT IV*, *β-1,3 glucanase*, *PR1* and *PR6* was observed at 24 h FET. It is known that PRs are defense proteins functioning in limiting pathogen multiplication and/or spread. Among the several metabolic alterations characteristic of HR, induction of PRs is a relatively late event (as previously reported), and their contribution to resistance against the initial infection is likely to be limited [46]. In *Bc*^−^/O_3_^+^ leaves, the genes involved in the cell wall degradation of fungi showed a transient over-expression (at 6 and 24 h FET in the case of *Chi IV* and *CHIT B*) or down-regulation throughout the whole period of the experiment (e.g., *β-1,3 glucanase*). Conversely, the genes involved in the detoxification of foreign compounds (e.g., *GST*) and in the recognition of virulence products (e.g., *PR1* and *PR6*) showed more persistent over-expression (except in the case of *PR6* at 24 h FET, *PR6* and *GST* at 48 h FET) confirming that O_3_ is able to activate at least two distinct signaling pathways, one of which overlaps with the HR and SAR activation pathways [18,47]. The examined genes categories seem to have reacted in different ways in leaves subjected to sequential double-treatments. In both *Bc*^+^ + O_3_^+^ and O_3_^+^ + *Bc*^+^ leaves, the expression of some genes is rapidly stimulated within a few hours after stress initiation (e.g., *CHIT B* and *GST* at 6 h FET; *CHIT IV*, *CHIT B*, *GST*, *PR1* and *PR6* at 12 h FET) indicating that they are involved in the early defence response [45]. Some of them showed more persistent over-expression at 24 (in the case of *CHIT IV*, *CHIT B*, *GST*, *PR1* and *PR6*) and 48 h FET (in the case of *PR1*) confirming their contribution to resistance against sequential double-treatments. It is worth to noting an additional up-regulation of *PR1* and *PR6* at 6 h FET only in *Bc*^+^ + O_3_^+^ leaves (as previously reported in *Bc*^+^/O_3_^−^ ones). In addition, a further over-regulation *CHIT B* (at 48 h FET) and *β-1,3 glucanase* (at 12 and 24 h FET as previously reported in *Bc*^−^/O_3_^+^ ones) was observed only in O_3_^+^ + *Bc*^+^ leaves indicating an overlap of *Bc*- and O_3_-mediated pathways [47]. All these mechanisms might be able to gradually shift the local defence response to a more systemic resistance [16].

**Figure 9:**
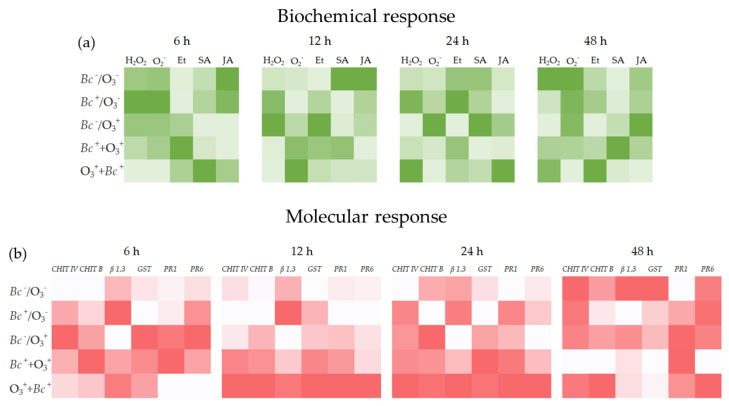
Heat maps of biochemical (**a**) and molecular (**b**) responses in leaves of *Vitis vinifera* var. Sangiovese uninoculated and maintained in filtered air (*Bc*^−^/O_3_^−^), inoculated with *Botrytis cinerea* and maintained in filtered air (*Bc*^+^/O_3_^−^), uninoculated with *B. cinerea* and treated with ozone (O_3_, 100 ppb, 3 h; *Bc*^−^/O_3_^+^), inoculated with *B. cinerea* and then subjected to O_3_ treatment (*Bc*^+^ + O_3_^+^), and treated with O_3_ and then inoculated with *B. cinerea* (O_3_^+^ + *Bc*^+^). Phytochemicals and gene expression level intensities were log_10_ transformed and are displayed as colours ranging from white to green (**a**) or red (**b**) at increasing intensities. Abbreviation: hydrogen peroxide, H_2_O_2_; anion superoxide, ^•^O_2_^−^; ethylene, Et; salicylic acid, SA; jasmonic acid, JA; chitinases B (*CHIT B*) and IV (*CHIT IV*); glutathione S-transferase, *GST*; β-1,3 glucanase, *β 1,3*; pathogenesis-related protein 1 (*PR1*) and 6 (*PR6*).

In response to the initial key questions, we can conclude that: first, the infection by *Bc* and O_3_ treatment *per se* share many similarities during the early stages after stress initiation including ROS production and HR-like PCD activation (Figure 9a). However, a divergence in ROS profiles and in the magnitude of their relative peaks was observed among leaves subjected to individual and sequential double-treatments. Second, several hormonal signaling cascades and in particular the balance between Et and SA (in *Bc*^+^/O_3_^−^ and *Bc*^+^ + O_3_^+^ leaves), Et and JA (in *Bc*^−^/O_3_^+^ samples), and Et-SA-JA (in *Bc*^−^/O_3_^+^ samples; Figure 9a) regulates the cell death program. Third, the examined genes categories seem to react in different ways in leaves subjected to individual and sequential double-treatments (Figure 9b) indicating different transcriptional responses are required for successful defense. Undoubtable, more studies are needed to better elucidate the involvement of signalling molecules at biochemical and genic level in response to multi-stress.

## 5. Conclusions

In conclusion, our study demonstrated the priming effects resulting from O_3_ treatment (100 ppb for 3 h) and inoculation with *Bc* in terms of protection against grey mold (preventive effect) and suppression of fungal inoculation (curative effect). These are fundamental goals in the development of emerging new techniques and novel methods to control fungal infections, by offering an alternative to the use of traditional chemicals for controlling one of the most important grapevine diseases. To the best of our knowledge, there are no studies on biochemical and molecular changes in processes/compounds related to SAR activation in *V. vinifera*-*Bc* pathosystem under O_3_ treatment. This is probably due to the fact that *Bc* infection constitutes a rare case of necrotrophic pathogen that induced SAR, but not always, it is not a rule. In addition, little information is available regarding the direct oxidative effect of O_3_ on pathogen structures.

Additional research is obviously required to evaluate the responses of other pathosystems to these effective and straightforward solutions, in order to control specific grapevine pathogens and elucidate the involvement of the signaling molecules at the biochemical and genic level.

## Data Availability

The data presented in this study are available in the article and Supplementary Materials.

## Supplementary Materials

The following supporting information can be downloaded at: https://www.mdpi.com/article/10.3390/antiox12020343/s1, Table S1: Gene name, forward and reverse sequence, GeneBank number access and efficiency (%) of primers used in RT-qPCR for gene expression analyses.
